# Finishing Performance, Meat Quality, and Economic Efficiency of Retired Thoroughbred Versus Belgian-Cross Geldings Under an Identical Total Mixed Ration: A Pilot Study

**DOI:** 10.3390/vetsci13030280

**Published:** 2026-03-18

**Authors:** Chanwool Park, Chansung Jeong, Miyeon Son, Junkoo Yi

**Affiliations:** 1 Gyeonggi Province Livestock Promotion Center, Gyeonggi-do Provincial Government, Suwon 16508, Republic of Korea; chamul90@gg.go.kr (C.P.); chanding@gg.go.kr (C.J.); 2 School of Animal Life Convergence Science, Hankyung National University, Anseong 17579, Republic of Korea; 3 Department of Food and Resource Economics, Kyungpook National University, Daegu 41566, Republic of Korea; 4 Gyeonggi Regional Research Center, Hankyong National University, Anseong 17579, Republic of Korea

**Keywords:** horse meat, off-the-track Thoroughbred, Belgian-crossbred, finishing, feed efficiency, carcass, intramuscular fat, economic feed conversion ratio

## Abstract

This study evaluated a 181-day finishing system for horses entering the Korean meat chain by comparing retired thoroughbred geldings and Belgian-crossbred geldings under identical management and an ad libitum forage-based total mixed ration. Ten geldings (*n* = 5 per group) were individually housed, with body weight and feed intake recorded monthly. After slaughter, carcass traits and meat quality grade were assessed, and longissimus thoracis et lumborum samples were analyzed for proximate composition. Belgian-crossbreds consumed more dry matter (18.68 vs. 13.60 kg DM/day), corresponding to 2.3% vs. 2.4% of body weight, but showed markedly greater growth (average daily gain 1.063 vs. 0.290 kg/day) and higher gain-to-feed (0.059 vs. 0.024) than retired Thoroughbreds. Carcass weight and marbling-related traits favored Belgian-crossbreds, including higher intramuscular fat in longissimus thoracis et lumborum (9.15% vs. 3.22%). Despite higher total feed cost per head, the economic feed conversion ratio was substantially lower in Belgian-crossbreds (13,133 vs. 35,088 KRW/kg gain), resulting in a positive gross margin estimate, whereas retired Thoroughbreds showed a negative margin under the same system. These results suggest that meat-type horses may be better suited to short, intensive finishing, while alternative utilization or tailored finishing strategies may be needed for retired racehorses.

## Simple Summary

Horses leaving racing in South Korea can enter the meat market, but it is unclear whether retired racehorses are efficient producers of meat. In this study, we compared retired Thoroughbred geldings with Belgian-crossbred geldings during a 181-day feeding period under the same conditions of the same housing and the same mixed diet with free access. Belgian-crossbred horses ate more, but gained weight much faster and produced heavier usable meat weight at slaughter. Their loin meat also contained more fat within the marbling, which can make the cooked meat juicier and more flavorful for consumers. Although the total feed cost per animal was higher for the Belgian-crossbreds, the feed cost needed to produce one kilogram of weight gain was much lower, giving a positive estimated profit. Retired Thoroughbreds showed the opposite trend. These results can help farmers choose suitable animals and feeding strategies, improving resource use and farm income.

## 1. Introduction

Horse meat is increasingly valued as a high-quality animal protein, and in several countries, it is produced as a differentiated livestock product with distinctive organoleptic characteristics [1,2,3]. In South Korea, however, a meaningful share of horse meat originates from horses leaving the racing industry, particularly off-the-track Thoroughbred geldings [4,5]. These animals are selected and trained for speed and endurance rather than for rapid fattening, yet they may enter finishing systems that were designed primarily for meat production [6,7]. Because retired racehorses are already present in the supply chain, evidence-based finishing strategies are needed to improve efficiency, product consistency, and economic returns while supporting the efficient and welfare-conscious utilization of these animals [4,8,9].

Many previous studies describing horse meat characteristics have compared breeds or horse types under different ages, diets, and management conditions [3,10,11,12,13,14,15]. Such comparisons are useful for general description but are harder to translate into on-farm decisions because production purpose and feeding intensity differ substantially among studies [3]. For finishing enterprises, the most actionable endpoints are those that determine profitability and marketability: dry matter intake, average daily gain, gain-to-feed ratio, carcass weight and yield, and marbling-related traits such as intramuscular fat [14,15,16]. Belgian-crossbred horses, which typically have greater mature size and draft-type influence than Thoroughbreds, represent a practical contrasting type that may better suit short, intensive finishing [6,17,18,19].

Therefore, the objective of this pilot study was to compare a 181-day fattening program and subsequent carcass and meat quality outcomes between off-the-track Thoroughbred geldings and Belgian-crossbred geldings maintained under the same management and fed the same total mixed ration [20,21,22]. As a pilot, we expected Belgian-crossbred geldings to show higher feed efficiency, more favorable carcass and meat quality traits, and improved feed-cost efficiency compared with off-the-track Thoroughbred geldings when finished under identical management and an identical total mixed ration. We focused on finishing performance, carcass traits, longissimus thoracis et lumborum meat quality (including proximate composition), and a feed-cost-based economic assessment [3,11,23,24]. By centering the analysis on the fattening period and outcomes most relevant to producers, this work aims to provide practical, preliminary evidence to guide breed-appropriate finishing decisions in the Korean horse meat sector [4,25]. Given the pilot sample size, these findings are presented as preliminary benchmarks under standardized management, and larger multi-site studies are warranted to confirm generalizability.

## 2. Materials and Methods

### 2.1. Animals and Finishing Design

Ten adult geldings were enrolled: off-the-track Thoroughbred geldings (*n* = 5; initial body mass 515.2 ± 17.3 kg; age 119.6 ± 21.8 months) and Belgian-crossbred geldings (*n* = 5; initial body mass 677.6 ± 24.3 kg; age 66.0 ± 2.4 months). Horses were finished for 181 days under the same management conditions. The study protocol was approved by the Institutional Animal Care and Use Committee of the Gyeonggi Province Livestock Promotion Center (Approval No. GPLP/001/21; approved on 14 June 2021).

### 2.2. Diet and Feeding Management

A forage-based total mixed ration was offered ad libitum throughout the 181-day finishing period [20,21]. The ration was formulated to meet the nutrient requirements for adult horses under a finishing (weight-gain) program as recommended by the National Research Council [26]. On a dry matter basis, the TMR contained 16.89% crude protein and an estimated 2.65 Mcal/kg metabolizable energy (estimated from TDN, 73.27% DM). Feed was supplied twice daily, and refusals were maintained to ensure free access [20,21]. The ingredient and chemical composition of the experimental ration are provided in Supplementary Tables S1 and S2.

### 2.3. Growth Performance and Feed Intake

Body weight was recorded monthly at a consistent time relative to feeding. Feed offering and refusal rates were measured to determine individual daily intake. Dry matter intake (DMI) was calculated from feed intake and the analyzed dry matter content. Average daily gain (ADG) was calculated as total body-weight gain divided by 181 days. Feed efficiency was expressed as gain-to-feed ratio (G/F: kg gain per kg dry matter intake) [16]. Monthly DMI and body-weight trajectories are provided in Supplementary Tables S3 and S5, and daily nutrient intakes are summarized in Supplementary Table S4. 

### 2.4. Blood Analysis (Health Monitoring)

To monitor physiological status during the finishing period, blood samples were collected at multiple time points and analyzed for serum biochemistry and hematology using commercial analyzers [27,28]. Full results are reported in Supplementary Table S6.

### 2.5. Slaughter, Carcass Traits, and Meat Quality Evaluation

Carcass weight was defined as hot carcass weight after exsanguination and removal of non-carcass components (i.e., excluding blood, hide, head, distal limbs, and visceral organs), according to the national grading standards (MAFRA Notice No. 2018-109) [29]. Dressing percentage was calculated as (carcass weight/pre-slaughter body mass) × 100. Retail-cut yields (20 cuts) are presented in Supplementary Table S7.

### 2.6. Proximate Composition and Detailed Meat Profiles

Longissimus thoracis et lumborum samples were analyzed for proximate composition (moisture, crude protein, crude fat, crude ash) [3,11]. Detailed profiles (minerals, amino acids, and fatty acids) are reported in Supplementary Tables S8–S10 [30,31,32,33].

### 2.7. Economic Evaluation

Economic performance was assessed from feed cost, cumulative weight gain, and economic feed conversion ratio (EFCR; KRW per kg gain). Total feed cost per head was calculated using the observed intake, ration unit price, and the 181-day feeding duration. EFCR was calculated as total feed cost divided by total weight gain. Estimated gross revenue and gross margin were calculated using a proxy willingness-to-pay value (26,961 KRW/kg gain) applied to live-weight gain [25,34,35]. The economic assessment was treated as a deterministic calculation based on observed intake and gain. To evaluate the sensitivity of gross margin to key assumptions, a one-way sensitivity analysis (±10% and ±20%) was conducted for the proxy willingness-to-pay value and for the TMR unit price; the results are provided in Supplementary Table S11.

### 2.8. Statistical Analysis

Normality was assessed using the Shapiro–Wilk test together with visual inspection of Q–Q plots, and the homogeneity of variances was evaluated using Levene’s test. Continuous outcomes were compared between groups using a two-sided Welch’s t-test when distributional assumptions were considered acceptable; otherwise, the Mann–Whitney U test was applied. Ordinal outcomes were analyzed using the Mann–Whitney U test. Statistical significance was defined as α = 0.05 (two-sided). Given the pilot sample size, inferential analyses were limited to prespecified primary endpoints, while monthly trajectories were presented descriptively. Analyses were performed using IBM SPSS Statistics, version 26.0 (IBM Corp., Armonk, NY, USA).

## 3. Results

### 3.1. Finishing Performance

**Figure 1:**
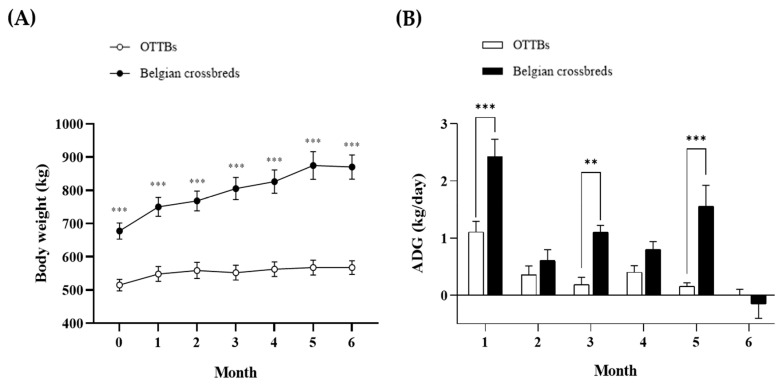
Monthly body weight and average daily gain during the 181-day finishing period in off-the-track Thoroughbred and Belgian-crossbred geldings. (**A**) Monthly body weight (Month 0–6). (**B**) Monthly average daily gain (Month 1–6). Open symbols/bars indicate off-the-track Thoroughbred geldings, and filled symbols/bars indicate Belgian-crossbred geldings. Data are presented as mean ± SEM (*n* = 5 per group). Asterisks denote between-group differences at the corresponding month (** *p* < 0.01; *** *p* < 0.001).

Belgian-crossbred geldings consumed more feed and exhibited substantially greater growth over the 181-day finishing period (Table 1 and Figure 1). Mean dry matter intake was higher in Belgian-crossbreds than in off-the-track Thoroughbreds (Supplementary Table S3). Consistent with this, Belgian-crossbreds showed markedly greater total weight gain and average daily gain, resulting in higher feed efficiency (gain-to-feed ratio) than off-the-track Thoroughbreds (Table 1). Monthly body-weight trajectories further demonstrated a clear separation between groups across the finishing period (Figure 1A), and monthly average daily gain was higher in Belgian-crossbreds (Figure 1B). Monthly intake and growth data are provided in Supplementary Tables S3 and S5.

### 3.2. Carcass Traits and Meat Quality

Carcass and meat quality outcomes favored Belgian-crossbreds (Table 2). Belgian-crossbreds produced heavier carcasses and showed a higher marbling score and a better overall meat quality grade than off-the-track Thoroughbreds. Under the national grading system used in this study, meat quality grade is assigned based on marbling and related quality attributes, with lower numerical grades indicating higher quality. Proximate composition of longissimus thoracis et lumborum supported these grading differences, with higher crude fat (intramuscular fat) and lower moisture in Belgian-crossbreds. The amino acid composition of the longissimus thoracis et lumborum is presented in Table 3 (units: g/100 g, wet weight). Overall, the profile was broadly comparable between groups, with similar totals of essential amino acids (8.514 ± 0.25 vs. 8.535 ± 0.22) and non-essential amino acids (9.136 ± 0.25 vs. 9.058 ± 0.22) in off-the-track Thoroughbreds and Belgian-crossbreds, respectively. Glutamic acid was the most abundant amino acid in both groups, consistent with its major contribution to meat flavor-related taste components, and methionine was modestly higher in off-the-track Thoroughbreds than in Belgian-crossbreds (*p* = 0.031; Table 4), while individual amino acids showed only small numerical differences. Detailed mineral and fatty acid profiles are provided in Supplementary Tables S8 and S10, and the full amino acid profile is provided in Supplementary Table S9.

### 3.3. Economic Efficiency

Although the total feed cost per head was higher for Belgian-crossbreds, the greater weight gain resulted in a lower EFCR compared with that of off-the-track Thoroughbreds (Table 4). Under the proxy willingness-to-pay assumption applied to live-weight gain, Belgian-crossbreds showed a positive estimated gross margin, whereas off-the-track Thoroughbreds showed a negative margin. In the sensitivity analysis (±10–20%), Belgian-crossbreds maintained a positive estimated gross margin across the tested ranges of willingness-to-pay and feed cost, whereas off-the-track Thoroughbreds remained negative or near break-even (Supplementary Table S11). This supports the robustness of the directional economic difference under plausible market variability.

## 4. Discussion

Under a standardized 181-day finishing system, clear differences emerged between off-the-track Thoroughbred geldings and Belgian-crossbred geldings in growth efficiency, carcass value, and economic outcomes. Belgian-crossbreds consumed more dry matter and achieved greater body-weight gain with a higher gain-to-feed ratio. From a finishing perspective, improved feed efficiency can reflect a shift in the maintenance-to-gain balance, whereby a greater proportion of intake is retained as tissue accretion rather than supporting maintenance requirements [7]. Given the pilot nature of the study, these results should be interpreted as preliminary benchmarks under the tested conditions rather than generalized estimates.

Carcass and meat quality responses were consistent with the performance results. Belgian-crossbreds produced heavier carcasses and showed more favorable marbling-related characteristics, including higher intramuscular fat in the longissimus thoracis et lumborum, compared with off-the-track Thoroughbreds. Intramuscular fat is widely recognized as a major driver of sensory attributes such as juiciness and flavor, and can influence consumer acceptance and value in markets where visible marbling is important [3,23]. One plausible physiological explanation for the higher feed efficiency observed in Belgian-crossbreds is a greater propensity for energy retention as adipose tissue during finishing, which can increase the fraction of intake allocated to gain relative to maintenance. Such a shift would be consistent with their higher intramuscular fat content and heavier carcasses under the same feeding system. Although our dataset is not designed to resolve mechanisms, this interpretation provides a biologically reasonable context for the observed pattern in this controlled comparison. Although marbling-related traits favored Belgian-crossbreds, off-the-track Thoroughbreds showed a larger longissimus muscle area and higher meat color scores (Table 2). This profile may reflect a comparatively leaner carcass phenotype and could be relevant for alternative product positioning depending on market preferences. In the present trial, routine health monitoring did not indicate overt metabolic disturbance, suggesting that the finishing regimen was practically implementable under the tested conditions [27,28]. All procedures were conducted under IACUC approval with routine health monitoring and ad libitum feeding, supporting welfare-compliant husbandry during the finishing period.

The economic assessment further supported these biological differences. Although daily and total feed costs per head were higher for Belgian-crossbreds, their much larger weight gain resulted in a substantially lower feed cost per kilogram of gain and a positive estimated gross margin under the applied assumptions, whereas off-the-track Thoroughbreds showed a negative margin. The gross-margin estimate depended on the proxy willingness-to-pay value used to monetize live-weight gain, but the direction of the difference between groups was consistent with the large divergence in feed efficiency [25]. For retired racehorses entering the meat supply chain, these results indicate that applying a uniform, short finishing strategy may be economically suboptimal. Alternative approaches may include extending the finishing duration, increasing dietary energy density, or positioning the product as a lean-meat option rather than aiming for high marbling within a short timeframe, consistent with prior evidence that finishing diet intensity and production system can materially affect carcass traits and meat composition in equids [14,15,26,35].

This pilot study is limited by the small sample size (*n* = 5 per group), restriction to a single farm and season, and the use of geldings from specific sources; therefore, its statistical power is limited, generalizability is restricted, and mechanistic inference should be avoided. Accordingly, we emphasize the direction and practical relevance of the between-group differences under standardized conditions, while acknowledging that confirmatory, multi-site studies are needed. Nevertheless, the standardized management and the consistent direction of effects across growth, carcass, meat quality, and economic endpoints provide practical preliminary evidence for improving decision-making in the Korean horse industry, where guidance on post-racing utilization and value-chain strategies remains an active topic [4]. More broadly, optimizing finishing strategies for different horse types may also contribute to sustainable meat production and product valorization, as highlighted in recent discussions of ecosystem services and gastronomic perceptions surrounding horse meat [8,9].

## 5. Conclusions

Under an identical 181-day finishing system, Belgian-crossbred geldings showed higher growth performance and feed efficiency than off-the-track Thoroughbred geldings. These differences were accompanied by heavier carcasses and more favorable marbling-related traits, including higher intramuscular fat in the loin muscle. Although total feed cost per head was higher for Belgian-crossbreds, feed cost per kilogram of gain was lower, and the estimated gross margin was positive under the assumptions applied, whereas off-the-track Thoroughbreds showed a negative margin in the same system. Overall, these findings provide preliminary, exploratory evidence that horse type may influence finishing efficiency, carcass value, and economic outcomes under standardized management, and that a single short, intensive finishing strategy may not be optimal for retired racehorses. Larger multi-site studies with greater statistical power are warranted to confirm these trends and to refine breed-appropriate finishing and marketing strategies.

## Figures and Tables

**Table 1:** Finishing performance of off-the-track Thoroughbred and Belgian-crossbred geldings during the 181-day finishing period.

Parameter	Off-the-Track Thoroughbreds (*n* = 5)	Belgian-Crossbreds (*n* = 5)	*p*-Value
Initial body weight (kg)	515.2 ± 17.3	677.6 ± 24.3	<0.001
Final body weight (kg)	567.6 ± 20.8	870.0 ± 36.3	<0.001
Total weight gain (kg)	52.4 ± 4.9	192.4 ± 21.7	0.002
Dry matter intake (kg DM/day)	13.6 ± 0.1	18.7 ± 0.1	<0.001
Dry matter intake (% of body weight) *	2.5	2.4	—
Average daily gain (kg/day)	0.290 ± 0.1	1.063 ± 0.1	0.002
Gain-to-feed ratio (kg gain/kg DM)	0.024 ± 0.1	0.059 ± 0.1	0.003

DM—dry matter. *p*-values are two-sided. Normality was assessed using the Shapiro–Wilk test with Q–Q plot inspection; homogeneity of variances was evaluated using Levene’s test. Continuous variables were compared using Welch’s t-test when assumptions were acceptable; otherwise, the Mann–Whitney U test was applied. Statistical significance was set at *p* < 0.05. Total weight gain was calculated at the individual-animal level (final minus initial body weight). * Dry matter intake (% BW) was calculated as (mean DMI, kg DM/day ÷ average body weight, kg) × 100, where average body weight was approximated as (initial + final body weight)/2; therefore, no *p*-value is reported for this derived ratio.

**Table 2:** Key carcass traits and meat quality outcomes of off-the-track Thoroughbred and Belgian-crossbred geldings.

Parameter	Off-the-Track Thoroughbreds (*n* = 5)	Belgian-Crossbreds (*n* = 5)	*p*-Value
Carcass traits and grading			
Carcass weight (kg)	373.0 ± 15.8	591.0 ± 29.0	<0.001
Carcass weight (% of final body mass) *	65.7	67.9	—
Backfat thickness (mm)	9.0 ± 1.7	10.0 ± 1.3	0.654
Longissimus muscle area (cm^2^)	106.0 ± 9.5	94.0 ± 9.2	0.391
Marbling score	1.0 ± 0.0	3.0 ± 0.7	0.046
Meat color score	5.6 ± 0.5	4.0 ± 0.0	0.033
Meat quality grade	3.0 ± 0.0	1.8 ± 0.4	0.040
Longissimus thoracis et lumborum proximate composition			
Moisture (%)	72.28 ± 0.3	67.65 ± 0.6	<0.001
Crude protein (%)	22.60 ± 0.4	21.21 ± 0.5	0.061
Crude fat/intramuscular fat (%)	3.22 ± 0.7	9.15 ± 1.1	0.003
Crude ash (%)	0.93 ± 0.1	0.87 ± 0.1	0.140

*p*-values are two-sided. Normality was assessed using the Shapiro–Wilk test with Q–Q plot inspection; homogeneity of variances was evaluated using Levene’s test. Continuous variables were compared using Welch’s t-test when assumptions were acceptable; otherwise, the Mann–Whitney U test was applied. Statistical significance was set at *p* < 0.05. Meat quality grade was assigned according to national grading standards; lower numerical grades indicate higher quality. * Carcass weight (% of final body mass) was calculated as (mean carcass weight, kg ÷ mean final body mass, kg) × 100 using group-level means; therefore, no *p*-value is reported for this derived ratio.

**Table 3:** Amino acid composition of longissimus thoracis et lumborum (g/100 g, wet weight).

Amino Acid/Total	Off-the-Track Thoroughbreds (*n* = 5)	Belgian-Crossbreds (*n* = 5)	*p*-Value
Arginine (Arg)	1.156 ± 0.04	1.147 ± 0.03	0.862
Leucine (Leu)	1.564 ± 0.05	1.572 ± 0.05	0.913
Lysine (Lys)	1.776 ± 0.06	1.775 ± 0.05	0.990
Methionine (Met)	0.549 ± 0.01	0.512 ± 0.01	0.031
Glutamic acid (Glu)	2.985 ± 0.09	2.966 ± 0.08	0.879
Alanine (Ala)	1.027 ± 0.03	1.048 ± 0.03	0.634
Glycine (Gly)	0.799 ± 0.02	0.830 ± 0.03	0.418
Total (Essential)	8.514 ± 0.25	8.535 ± 0.22	0.951
Total (Non-essential)	9.136 ± 0.25	9.058 ± 0.22	0.821

Units are g/100 g (wet weight). *p*-values are two-sided and were calculated using Welch’s *t*-test (α = 0.05), consistent with the Statistical Analysis section. The complete amino acid profile is provided in Supplementary Table S9.

**Table 4:** Economic efficiency of the 181-day finishing system.

Parameter	Off-the-Track Thoroughbreds (*n* = 5)	Belgian-Crossbreds (*n* = 5)	*p*-Value
TMR unit price (KRW/kg, as-fed)	654.6	654.6	-
Daily feed cost (KRW/head/day)	10,158	13,960	<0.001
Total feed cost (KRW/head; 181 days)	1,838,626	2,526,823	<0.001
Total weight gain (kg/head)	52.4	192.4	0.002
EFCR (KRW/kg gain)	35,088	13,133	0.001
Estimated gross revenue (KRW/head)	1,412,756	5,187,296	-
Estimated gross margin (KRW/head)	−425,870	+2,660,473	-

Estimated gross revenue and gross margin were calculated using a proxy willingness-to-pay value of 26,961 KRW per kg live-weight gain applied to observed weight gain; these are deterministic estimates and were not subjected to hypothesis testing. EFCR was calculated at the individual-animal level (total feed cost divided by total weight gain per horse) and compared between groups using the same parametric/nonparametric decision rules described in Table 1 (α = 0.05, two-sided). If a sensitivity analysis is included, it can be found in Supplementary Table S11.

## Data Availability

The data presented in this study are available upon request from the corresponding author due to institutional restrictions on data sharing at the Gyeonggi Province Livestock Promotion Center.

## Supplementary Materials

The following supporting information can be downloaded at https://www.mdpi.com/article/10.3390/vetsci13030280/s1: Table S1: Ingredient composition of the experimental total mixed ration (TMR); Table S2: Chemical composition of the experimental TMR (dry matter basis); Table S3: Monthly dry matter intake (DMI) and gain-to-feed ratio (G/F) during the finishing period; Table S4: Daily nutrient intake during the finishing period; Table S5: Monthly body weight and average daily gain (ADG) during the finishing period; Table S6: Serum biochemistry and hematology indices during finishing (health monitoring); Table S7: Retail-cut yields (20 cuts) and part-wise carcass distribution; Table S8: Full proximate composition and mineral content of longissimus thoracis et lumborum; Table S9: Amino acid composition of longissimus thoracis et lumborum (g/100 g, wet weight); Table S10: Fatty acid composition of longissimus thoracis et lumborum; Table S11: One-way sensitivity analysis (±10% and ±20%) for gross margin estimates based on proxy willingness-to-pay and TMR unit price.
