# Supplementary material for: Finishing Performance, Meat Quality, and Economic Efficiency of Retired Thoroughbred Versus Belgian-Cross Geldings Under an Identical Total Mixed Ration: A Pilot Study
Source: Vet Sci. 2026 Mar 18;13(3):280. doi: 10.3390/vetsci13030280 (PMC13029992; doi:10.3390/vetsci13030280)
Supplement: Supplementary file 1 [file vetsci-13-00280-s001.zip › vetsci-4129857-supplementary.pdf]

Supplementary Materials

Table S1. Ingredient composition of the experimental total mixed ration (TMR).

Ingredient	DM basis (%)
corn-based concentrate pellet 1)	30.437
Alfalfa hay	9.818
Timothy hay	9.818
Whole oat grain	8.837
Molasses	6.382
Alfalfa pellet	4.909
Oat grain pellet	4.909
Corn gluten feed	4.418
Corn germ meal	3.927
TMR base mixture 2)	3.927
Steam-flaked corn	2.946
Steam-flaked lupin	2.946
Soybean meal	2.946
Oat	2.209
Premix vitamin	0.491
Salt (NaCl)	0.393
Sodium bicarbonate 3)	0.295
Limestone (Calcium carbonate, $CaCO_3$)	0.196
Amino acid supplement	0.196
Total	100.00

All values are expressed on a dry matter (DM) basis. Totals may differ slightly from 100% due to rounding.

1) The diet included corn, corn gluten, corn DDGS, and a molasses-blended pellet. The molasses-blended pellet had the following composition on a dry matter basis: DM 89.6%, CP 20.00%, EE 5.44%, CF 13.77%, Ca 0.78%, P 0.66%, ADF 18.10%, NDF 37.50%, and TDN 73.00%.

2) The OEM concentrate feed had the following composition on a dry matter basis: DM 45.00%, CP 6.50%, CF 7.00%, ADF 9.00%, and NDF 12.00%.

3) The feed contained per kilogram: vitamin A, 2,000,000 IU; vitamin D_3, 400,000 IU; and vitamin E, 4,000 IU.

Table S2. Chemical composition of the experimental TMR (dry matter basis).

Chemical Component	Value (% DM)
Dry matter	87.61
Crude protein	16.89
Crude fat (ether extract)	2.63
Crude fiber	18.01
Crude ash	10.49
Calcium	0.40
Phosphorus	0.92
NDF	45.75
ADF	25.47
TDN	73.27

Values are expressed on a dry matter basis.

Table S3. Monthly dry matter intake (DMI) and gain-to-feed ratio (G:F) during the finishing period.

(a) Monthly DMI (kg/day)

Breed \ Month	1	2	3	4	5	6	Overall mean
OTTBs	12.27 ± 0.26	14.92 ± 0.52	15.94 ± 0.30	13.27 ± 0.09	13.94 ± 0.14	11.24 ± 0.14	13.60 ± 0.11
Belgian crossbreds	20.69 ± 0.60	21.59 ± 0.42	19.33 ± 0.42	16.77 ± 0.07	16.73 ± 0.08	17.00 ± 0.08	18.68 ± 0.11

(b) G:F (kg gain/kg feed)

Breed \ Month	1	2	3	4	5	6	Overall mean
OTTBs	0.090 ± 0.016	0.024 ± 0.010	−0.012 ± 0.008	0.030 ± 0.009	0.011 ± 0.004	0.000 ± 0.009	0.024 ± 0.002
Belgian crossbreds	0.117 ± 0.016	0.028 ± 0.008	0.057 ± 0.005	0.048 ± 0.008	0.093 ± 0.022	−0.009 ± 0.015	0.059 ± 0.006

Values are presented as mean ± SEM; n = 5 per breed. (a) DMI is expressed as kg/day. (b) G:F was calculated as average daily gain (kg/day) divided by DMI (kg/day).

Table S4. Daily nutrient intake during the finishing period.

Breed / Intake	OTTBs	Belgian crossbreds	p-value
DMI	13.60	18.68	< 0.001
TDN	9.96	13.70	< 0.001
Crude protein	2.230	3.15	< 0.001
Ca	0.05	0.07	< 0.001
P	0.12	0.17	< 0.001
ADF	3.46	4.76	< 0.001
NDF	6.22	8.55	< 0.001

All values are expressed as kg/head/day on a dry matter basis (means over the 181-day period).

Table S5. Monthly body weight and average daily gain (ADG) during the finishing period.

(a) Monthly body weight (kg)

Month / Breed	Start	1	2	3	4	5	6	Final	Overall mean
OTTBs	515.2 ± 17.27	548.4 ± 22.37	558.8 ± 24.25	552.4 ± 22.16	562.8 ± 21.81	567.6 ± 22.29	567.6 ± 20.79	567.6 ± 20.79	559.6
Belgian crossbreds	677.6 ± 24.28	750.4 ± 28.42	768.0 ± 29.80	805.4 ± 33.19	826.4 ± 35.01	874.8 ± 41.52	870.0 ± 36.26	870.0 ± 36.26	815.8

(b) Monthly ADG (kg/day)

Month / Breed	1	2	3	4	5	6	Overall mean
OTTBs	1.107 ± 0.185	0.359 ± 0.153	−0.188 ± 0.123	0.400 ± 0.115	0.155 ± 0.060	0.000 ± 0.102	0.290 ± 0.027
Belgian crossbreds	2.427 ± 0.303	0.607 ± 0.189	1.106 ± 0.115	0.800 ± 0.135	1.561 ± 0.362	-0.155 ± 0.250	1.063 ± 0.120

Values are presented as mean ± SEM; n = 5 per breed. (a) Start indicates baseline body weight at day 0; Final indicates body weight at day 181. (b) Monthly ADG (kg/day) was calculated as the difference in body weight between two consecutive measurements divided by the number of days separating them. The overall mean for ADG is the arithmetic mean of monthly ADG across six months.

Table S6. Serum biochemistry and hematology indices during finishing (health monitoring).

(a) Serum biochemistry parameters

Parameter	OTTBs	Belgian crossbreds	Reference range	p-value
GLU (mg/dL)	88.35 ± 3.73	89.7 ± 3.49	64 – 150	0.793
CREA (mg/dL)	1.01 ± 0.04	1.05 ± 0.06	0.8 – 2.2	0.562
BUN (mg/dL)	14.30 ± 0.07	16.50 ± 0.41	10 – 25	0.010
GGT (U/L)	17.40 ± 1.47	30.70 ± 4.81	0 – 87	0.015
CK (U/L)	121.80 ± 12.16	190.72 ± 40.67	10 – 350	0.098
TP (g/dL)	6.12 ± 0.22	6.93 ± 0.21	5.6 – 7.9	0.012
ALB (g/dL)	3.17 ± 0.12	3.42 ± 0.12	1.9 – 3.2	0.154
GLOB (g/dL)	2.97 ± 0.11	3.52 ± 0.11	2.4 – 4.7	0.001
TBIL (g/dL)	1.06 ± 0.09	0.78 ± 0.08	0.0 – 3.5	0.020
ALT (U/L)	43.20 ± 1.71	57.65 ± 3.04	5 – 50	<0.001
LDH (U/L)	630.15 ± 39.25	1112.89 ± 106.86	250 – 2070	<0.001

Values are presented as mean ± SEM; n = 5 per breed. Reference ranges are manufacturer-provided for adult horses (Catalyst One chemistry analyzer) (IDEXX Laboratories Inc., 2024).

(b) Hematology parameters

Parameter	OTTBs	Belgian crossbreds	Reference range	p-value
WBC (K/μL)	6.69 ± 0.25	7.69 ± 0.25	4.9 – 11.1	0.007
PLT (K/μL)	119.00 ± 5.66	157.30 ± 9.39	100 – 250	0.001
LYM (K/μL)	2.58 ± 0.16	3.51 ± 0.20	1.5 – 5.1	0.001
HGB (g/dL)	13.61 ± 0.32	15.28 ± 0.31	1.7 – 16.5	0.001
MCHC (g/dL)	35.02 ± 0.15	34.27 ± 0.11	32.8 – 38.6	<0.001
RBC (M/μL)	8.29 ± 0.22	9.63 ± 0.16	6.4 – 10.4	<0.001
HCT (%)	38.88 ± 0.93	44.60 ± 0.89	30 – 47	<0.001
MCV (fL)	46.94 ± 0.43	46.31 ± 0.44	41.1 – 52.4	0.313
MCH (pg)	16.43 ± 0.14	15.87 ± 0.18	14.1 – 18.6	0.016

Values are presented as mean ± SEM; n = 5 per breed. Reference ranges are manufacturer-provided for adult horses (ProCyte Dx hematology analyzer) (IDEXX Laboratories Inc., 2021).

Table S7. Retail-cut yields (20 cuts) and part-wise carcass distribution.

Carcass Part	OTTBs		Belgian crossbreds	
	Weight (kg)	Yield (%)	Weight (kg)	Yield (%)
Longissimus thoracis et lumborum	26.3 ± 1.7	10.8 ± 0.2	33.2 ± 2.2	10.2 ± 0.3
Tenderloin	8.7 ± 0.6	3.6 ± 0.1	10.0 ± 0.5	3.1 ± 0.1
Chuck	28.0 ± 1.8	11.6 ± 0.2	31.4 ± 3.2	9.8 ± 1.1
Top round	26.6 ± 1.6	11.0 ± 0.2	31.1 ± 1.7	9.6 ± 0.4
Bottom round	56.1 ± 3.5	23.1 ± 0.2	70.9 ± 4.3	21.8 ± 0.6
Brisket	15.7 ± 1.0	6.5 ± 0.1	19.6 ± 0.8	6.0 ± 0.1
Hanging tender	0.9 ± 0.1	0.4 ± 0.0	0.8 ± 0.1	0.3 ± 0.0
Outside skirt	0.6 ± 0.1	0.2 ± 0.0	0.6 ± 0.0	0.2 ± 0.0
Chuck roll	18.5 ± 1.6	7.6 ± 0.3	23.0 ± 1.2	7.1 ± 0.3
Inside skirt	3.6 ± 0.1	1.5 ± 0.1	4.7 ± 0.3	1.4 ± 0.1
Short plate	13.2 ± 0.9	5.4 ± 0.1	14.7 ± 1.1	4.5 ± 0.2
Flank steak	4.2 ± 0.2	1.8 ± 0.1	5.9 ± 0.7	1.8 ± 0.2
Ribs	39.7 ± 1.5	16.5 ± 0.6	78.8 ± 6.2	24.2 ± 1.0
Shank	17.5 ± 1.1	7.2 ± 0.0	20.5 ± 1.2	6.3 ± 0.2
Striploin	13.3 ± 0.7	5.5 ± 0.1	16.6 ± 1.0	5.1 ± 0.1
tendon	1.8 ± 0.1	0.8 ± 0.1	2.7 ± 0.2	0.8 ± 0.1
knuckle	1.4 ± 0.1	0.6 ± 0.0	2.2 ± 0.1	0.7 ± 0.0
tail	10.6 ± 0.5	4.4 ± 0.2	15.5 ± 0.4	4.8 ± 0.2
Marrow bone	21.7 ± 1.0	9.0 ± 0.2	29.0 ± 1.3	9.0 ± 0.4
Trimmed bones	20.8 ± 0.8	8.6 ± 0.4	26.8 ± 1.2	8.3 ± 0.3
Total (horse meat)	241.9 ± 13.8	100.0	324.7 ± 15.7	100.0

Values are presented as mean ± SEM; n = 5 per breed. Yields are percent of total horse meat in this table (total = 100%). Some cuts may overlap; therefore, row percentages may not sum to 100%.

Table S8. Full proximate composition and mineral content of longissimus thoracis et lumborum.

Component	OTTBs	Belgian crossbreds
Moisture (%)	72.28 ± 0.32	67.65 ± 0.58
Crude Protein (%)	22.60 ± 0.40	21.21 ± 0.49
Crude fat (%)	3.22 ± 0.74	9.15 ± 1.11
Crude Ash (%)	0.93 ± 0.03	0.87 ± 0.02
Ca (mg/kg)	40.90 ± 1.56	39.35 ± 0.60
Cu (mg/kg)	0.92 ± 0.14	0.15 ± 0.04
Fe (mg/kg)	34.29 ± 5.31	19.71 ± 0.37
K (mg/kg)	3297.70 ± 87.23	3206.62 ± 47.77
Mg (mg/kg)	224.49 ± 5.35	216.57 ± 6.29
Mn (mg/kg)	ND	ND
Na (mg/kg)	386.79 ± 21.85	378.81 ± 5.58
P (mg/kg)	2341.99 ± 36.56	1893.58 ± 84.45
Zn (mg/kg)	28.77 ± 2.07	31.42 ± 2.14

All values are presented as mean ± SEM; n = 5 per breed. ND: not detected.

Table S9. Amino acid composition of longissimus thoracis et lumborum.

Amino acid	OTTBs	Belgian crossbreds
**Conditionally essential amino acid**		
Arginine (Arg)	1.156 ± 0.04	1.147 ± 0.03
**Essential amino acids**		
Threonine (Thr)	0.911 ± 0.03	0.900 ± 0.02
Valine (Val)	1.015 ± 0.03	1.050 ± 0.03
Methionine (Met)	0.549 ± 0.01	0.512 ± 0.01
Isoleucine (Ile)	0.968 ± 0.03	0.986 ± 0.03
Leucine (Leu)	1.564 ± 0.05	1.572 ± 0.05
Phenylalanine (Phe)	0.850 ± 0.02	0.844 ± 0.02
Histidine (His)	0.882 ± 0.04	0.896 ± 0.04
Lysine (Lys)	1.776 ± 0.06	1.775 ± 0.05
Total (Essential)	8.514 ± 0.25	8.535 ± 0.22
**Non-essential amino acids**		
Aspartic acid (Asp)	1.791 ± 0.05	1.815 ± 0.05
Serine (Ser)	0.777 ± 0.02	0.732 ± 0.02
Glutamic acid (Glu)	2.985 ± 0.09	2.966 ± 0.08
Glycine (Gly)	0.799 ± 0.02	0.830 ± 0.03
Alanine (Ala)	1.027 ± 0.03	1.048 ± 0.03
Tyrosine (Tyr)	0.817 ± 0.02	0.741 ± 0.04
Proline (Pro)	0.646 ± 0.02	0.649 ± 0.03
Cysteine (Cys)	0.294 ± 0.00	0.277 ± 0.01
Total (Non-essential)	9.136 ± 0.25	9.058 ± 0.22

Values are presented as mean ± SEM; units are g/100 g (wet weight). n = 5 per breed.

Table S10. Fatty acid composition of longissimus thoracis et lumborum.

Fatty acid	OTTBs	Belgian crossbreds
Myristic acid (C14:0)	5.80 ± 0.12	5.13 ± 0.26
Palmitic acid (C16:0)	30.70 ± 0.52	32.40 ± 0.29
Palmitoleic acid (C16:1n-7)	7.77 ± 0.37	11.72 ± 0.39
Stearic acid (C18:0)	4.12 ± 0.13	3.38 ± 0.23
Oleic acid (C18:1n-9)	35.41 ± 0.48	38.58 ± 0.69
Vaccenic acid (C18:1n-7)	ND	ND
Linoleic acid (C18:2n-6)	13.11 ± 0.71	7.11 ± 0.35
γ-linolenic acid (GLA, C18:3n-6)	0.03 ± 0.00	0.02 ± 0.00
α-Linolenic acid(ALA, C18:3n-3)	2.01 ± 0.19	0.92 ± 0.03
Eicosenoic acid (C20:1n-9)	0.57 ± 0.02	0.47 ± 0.02
Arachidonic acid (C20:4n-6)	0.47 ± 0.03	0.27 ± 0.04
Eicosapentaenoic acid (EPA, C20:5n-3)	ND	ND
DHA(C22:6n-3)	ND	ND
Docosatetraenoic acid (C22:4n-6)	ND	ND
Saturated fatty acids (SFA)	40.62 ± 0.48	40.92 ± 0.30
Monounsaturated fatty acids (MUFA)	43.76 ± 0.76	50.77 ± 0.62
Polyunsaturated fatty acids (PUFA)	15.62 ± 0.85	8.31 ± 0.40
Total	100.000	100.000

Values are presented as mean ± SEM; n = 5 per breed. Expressed as % of total identified fatty acids. Totals may differ slightly from 100% due to rounding. ND: not detected.

Table S11A. Sensitivity analysis of gross margin to the proxy willingness-to-pay value (±10–20%).

WTP change	WTP (KRW/kg gain)	Gross margin (Thoroughbred)	Gross margin (Begian crossbred)
−20%	21,568.8	−708,421	1,623,014
−10%	24,264.9	−567,145	2,141,744
0%	26,961.0	−425,870	2,660,473
+10%	29,657.1	−284,594	3,179,203
+20%	32,353.2	−143,318	3,697,933

Unit: KRW/head; base-case uses willingness-to-pay = 26,961 KRW/kg live-weight gain.

Table S11B. Sensitivity analysis of gross margin to feed cost (±10–20% change in total feed cost).

Feed cost change	Total feed cost (Thoroughbred)	Gross margin (Thoroughbred)	Total feed cost (Belgian-crossbred)	Gross margin (Begian crossbred)
−20%	1,470,901	−58,144	2,021,458	3,165,838
−10%	1,654,763	−242,007	2,274,141	2,913,156
0%	1,838,626	−425,870	2,526,823	2,660,473
+10%	2,022,489	−609,732	2,779,505	2,407,791
+20%	2,206,351	−793,595	3,032,188	2,155,109

Unit: KRW/head; revenue fixed at base-case willingness-to-pay.
